# A nomogram for predicting the risk of poor prognosis in patients with poor-grade aneurysmal subarachnoid hemorrhage following microsurgical clipping

**DOI:** 10.3389/fneur.2023.1146106

**Published:** 2023-03-22

**Authors:** Zhaopeng Zhou, Zhuanghua Liu, Hongqiao Yang, Chunlei Zhang, Chenxu Zhang, Junhui Chen, Yuhai Wang

**Affiliations:** Department of Neurosurgery, The 904th Hospital of Joint Logistic Support Force of PLA, Wuxi Clinical College of Anhui Medical University, Wuxi, Jiangsu, China

**Keywords:** subarachnoid hemorrhage (SAH), aneurysm, poor-grade, outcome, nomogram

## Abstract

**Objective:**

Aneurysmal subarachnoid hemorrhage (aSAH) is a common and potentially fatal cerebrovascular disease. Poor-grade aSAH (Hunt-Hess grades IV and V) accounts for 20–30% of patients with aSAH, with most patients having a poor prognosis. This study aimed to develop a stable nomogram model for predicting adverse outcomes at 6 months in patients with aSAH, and thus, aid in improving the prognosis.

**Method:**

The clinical data and imaging findings of 150 patients with poor-grade aSAH treated with microsurgical clipping of intracranial aneurysms on admission from December 2015 to October 2021 were retrospectively analyzed. Least absolute shrinkage and selection operator (LASSO), logistic regression analyses, and a nomogram were used to develop the prognostic models. Receiver operating characteristic (ROC) curves and Hosmer–Lemeshow tests were used to assess discrimination and calibration. The bootstrap method (1,000 repetitions) was used for internal validation. Decision curve analysis (DCA) was performed to evaluate the clinical validity of the nomogram model.

**Result:**

LASSO regression analysis showed that age, Hunt-Hess grade, Glasgow Coma Scale (GCS), aneurysm size, and refractory hyperpyrexia were potential predictors for poor-grade aSAH. Logistic regression analyses revealed that age (*OR*: 1.107, 95% *CI*: 1.056–1.116, *P* < 0.001), Hunt-Hess grade (*OR*: 8.832, 95% *CI*: 2.312–33.736, *P* = 0.001), aneurysm size (*OR*: 6.871, 95% *CI*: 1.907–24.754, *P* = 0.003) and refractory fever (*OR*: 3.610, 95% *CI*: 1.301–10.018, *P* < 0.001) were independent predictors of poor outcome. The area under the ROC curve (AUC) was 0.909. The calibration curve and Hosmer–Lemeshow tests showed that the nomogram had good calibration ability. Furthermore, the DCA curve showed better clinical utilization of the nomogram.

**Conclusion:**

This study provides a reliable and valuable nomogram that can accurately predict the risk of poor prognosis in patients with poor-grade aSAH after microsurgical clipping. This tool is easy to use and can help physicians make appropriate clinical decisions to significantly improve patient prognosis.

## 1. Introduction

Subarachnoid hemorrhage (SAH) is a common and potentially fatal cerebrovascular disease. Ruptured intracranial aneurysms are the leading cause of morbidity in 80% of the patients with SAH. As a subtype of stroke, it accounts for 2–7% of strokes. It has an annual global incidence of 6–9 people per 100,000 (1–3). SAH is associated with short-term prognostic mortality rates of 8.3–66.7% have been reported for SAH, with reasonable prognosis rates of only 36–55% (4). Consequently, patients with SAH have long treatment cycles and high costs, which impose a severe economic burden on their families and the society (5).

Poor-grade aneurysm SAH (aSAH), classified as grade IV or V by the Hunt-Hess grade, accounts for 20–30% of patients, and most of these patients have a poor prognosis (6). Ruptured intracranial aneurysms can be managed by surgical clipping or endovascular coiling. However, management of complications caused by early brain injury (EBI) and delayed brain injury (DBI) after surgery remains difficult (7). Therefore, it is essential to develop early predictive models for poor prognosis for the clinical management of patients with poor-grade aSAH.

Risk prediction models can predict outcomes based on highly influential indicators obtained from the medical history, physical examination, laboratory tests, and radiology, thereby contributing significantly to the decision-making process for the clinical management of patients. Establishing a nomogram using traditional least absolute shrinkage and selection operator (LASSO) and logistic regression analysis remains the primary approach for developing clinical disease prediction models that are widely used in diagnosing or predicting the progression of diseases through a combination of multiple indicators (8). A reliable predictive model is essential for physicians to make appropriate clinical decisions that can significantly improve patient prognosis. Recent studies have successfully predicted the long-term prognosis and analyzed the utility of management strategies after SAH (6, 9, 10). Unfortunately, few studies have focused on the role of clinical information in predicting the clinical outcomes of patients with poor-grade aSAH after surgical clipping.

In this study, we retrospectively analyzed the clinical data of patients with poor-grade aSAH who underwent surgical clipping. This study aimed to develop a stable nomogram model for predicting adverse outcomes at 6 months in this patient population, and help improve the prognosis.

## 2. Materials and methods

### 2.1. Study population

We retrospectively analyzed the medical records of 150 patients with poor-grade aSAH admitted to the Department of Neurosurgery of the 904th Hospital of Joint Logistic Support Force of PLA between December 2015 and October 2021, who were initially treated with microsurgical clipping of intracranial aneurysms. This retrospective study conformed to the principles of the Declaration of Helsinki and, did not violate the code of ethics; patients or their families signed informed consent forms prior to treatment.

The inclusion criteria were as follows: (1) age ≥ 18 years; (2) diagnosis of a ruptured intracranial aneurysm resulting in SAH confirmed by CT angiography (CTA) or digital subtraction angiography (DSA) on admission; (3) Hunt-Hess grades IV and V; and (4) surgical clamping performed for a ruptured aneurysm. The exclusion criteria were as follows: (1) age < 18 years; (2) Hunt-Hess grade I–III; (3) diagnosis of spontaneous subarachnoid hemorrhage without further cranial CTA or DSA; (4) death or patient loss to follow-up; (5) combined traumatic SAH or other bleeding disorders; and (6) multiple organ failure.

### 2.2. Collection of clinical information

All patients admitted to our hospital on an emergency basis underwent early resuscitation and CTA. The decision to perform surgical clipping was determined by a multidisciplinary team of neurosurgeons and anesthesiologists based on the clinical situation and consent of the family members. Microsurgical clipping: if the intracerebral hematoma has a significant occupying effect and/or is combined with acute hydrocephalus, or if the family has a strong desire for treatment, the aneurysm should be surgically clamped. Routine treatments, such as anti-inflammatory, hemostatic, and analgesic drugs, were administered throughout hospitalization according to the aSAH management guidelines (11). Postoperative cranial CT was performed to determine the presence of intracranial rebleeding or cerebral infarction. The following clinical and imaging findings of the patients were collected: (1) preoperative clinical data, including age, sex, previous history (hypertension and diabetes), Hunt-Hess grade, Fisher grade, Glasgow Coma Scale (GCS) score, aneurysm size, number of aneurysms and location, whether the patient had any physical impairment, dilated pupils, and intracranial hematoma; (2) surgical data: operative time from the onset of aSAH to surgery (ultra-early stage: < 8 h; early stage:8–24 h; mid-late stage: >24 h), intraoperative encephalocele; and (3) postoperative complications, including refractory fever, cerebral vascular spasm (CVS), hydrocephalus, cerebral infarction, intracranial infection, and epilepsy. Refractory fever was defined as when the patient's temperature exceeded 38.3°C, simple physical and pharmacological cooling failed, and the condition of fever was of unknown etiology. CVS refers to the narrowing of the lumen of the cerebral vessels of the internal carotid artery or vertebrobasilar system. Transcranial Doppler (TCD) ultrasonography is commonly used clinically to predict CVS, which is diagnosed when the middle cerebral artery blood flow velocity is >120 cm/s.

### 2.3. Outcome data

Patient's prognosis was assessed using face-to-face measurements of the GOS by neurosurgeons more than 6 months after surgery. The GOS score for this assignment was as follows: (1) 5 points: good recovery, return to normal life despite mild impairment; (2) 4 points: minor disability but can live independently and can work under protection; (3) 3 points: severe disability awake, handicapped, and in need of daily care; (4) 2 points: minimally responsive vegetative survival (e.g., eyes open during sleep or wake cycle); and (5) 1 point: death. Patients with a GOS score > 3 were considered to have a good prognosis and ≤ 3 were considered to have a poor prognosis.

### 2.4. Statistical analyses

SPSS 26.0 statistical software (SPSS Inc., Chicago, USA) and R version 4.1.2 (R) was used for statistical analyses. Continuous variables were expressed as mean ± standard deviation, and count variables were expressed as rate (%). Differences in clinical characteristics between the two groups were assessed using the chi-square tests, *t*-test, Fisher's exact test, and Wilcoxon rank-sum test. In addition, features with non-zero coefficients in the LASSO regression model were used as candidate predictors of poor prognosis using the LASSO regression method, which is suitable for small samples and high-dimensional data. LASSO regression can achieve variable selection and model parameter estimation simultaneously, which can better solve the problem of multicollinearity in regression analysis and can explain the results well. Subsequently, these factors were used to identify risk factors affecting poor prognosis, and were included in univariate and multifactor logistic regression analyses. A predictive nomogram was created by assigning each significant risk factor an initial score on the graph ranging from 0 to 10.

Receiver operating characteristic (ROC) curve analysis was performed to assess the discrimination of the prediction model. The Hosmer–Lemeshow test was used to assess the goodness of fit of the nomogram (*P* > 0.05, indicating a good fit of the nomogram). The bootstrap method (1,000 repetitions) was used for internal validation. Nomogram performance was evaluated using Harrell's consistency index (C-index) and calibration plots. A c-index > 0.7 reflects a good fitting characteristic of the prediction model. Finally, decision curve analysis (DCA) was performed to evaluate the clinical validity of the nomogram model. A *P*-value < 0.05 was considered statistically significant.

## 3. Results

### 3.1. Baseline characteristics

A total of 150 patients with poor-grade aSAH who underwent aneurysm clipping were enrolled in the current study, including 64 with good outcomes (42.7%) and 86 with poor prognoses (57.3%). As shown in Table 1, there were no significant differences (*P* > 0.05) in sex, diabetes mellitus, multiple aneurysms, aneurysm location, surgical timing, physical impairment, intracranial hematoma, hydrocephalus, CVS, intracranial infection or epilepsy between the good and poor outcome groups. There were obvious significant differences (*P* < 0.05) in age (*t* = −3.893, *P* < 0.001), GCS (*t* = 6.770, *P* < 0.001), Hunt-Hess grade (χ^2^ = 41.148, *P* < 0.001), Fisher grade (χ^2^ = 41.440, *P* = 0.032), hypertension (χ^2^ = 4.642, *P* = 0.031), aneurysm size (χ^2^ = 17.068, *P* < 0.001), pupillary dilation (χ^2^ = 46.243, *P* = 0.012), encephalocele (χ^2^ = 11.192, *P* = 0.001), cerebral infarction (χ^2^ = 47.181, *P* = 0.007), and refractory fever (χ^2^ = 21.366, *P* < 0.001) between the two groups.

**Table 1:** Baseline characteristics.

**Variables**	**Total (*n* = 150)**	**Good outcome (*n* = 64)**	**Poor outcome (*n* = 86)**	**Test value**	***P*-value**
Sex [*n* (%)]				3.648	0.056
Female	85 (56.7)	42 (65.6)	43 (50.0)		
Male	65 (43.3)	22 (34.4)	43 (50.0)		
Age (Mean ± SD)	55 ± 11.500	51.80 ± 10.594	58.88 ± 11.274	−3.893	< 0.001
GCS (Mean ± SD)	5.52 ± 1.996	6.67 ± 1.968	4.66 ± 1.539	6.770	< 0.001
Hunt-Hess grade				41.148	< 0.001
IV	74 (49.3)	51 (79.7)	23 (26.7)		
V	76 (50.7)	13 (20.3)	63 (73.3)		
Fisher grade				4.440	0.032
1–3	26 (17.3)	16 (25.0)	10 (11.6)		
4	124 (82.7)	48 (75.0)	76 (88.4)		
Hypertension				4.642	0.031
+	118 (78.7)	45 (70.3)	73 (84.9)		
–	32 (21.3)	19 (29.7)	13 (15.1)		
Diabetes mellitus				1.093	0.296
+	15 (10.0)	4 (6.3)	11 (12.8)		
–	135 (90.0)	60 (93.8)	75 (87.2)		
Aneurysm size				17.068	< 0.001
< 10 mm	110 (73.3)	58 (90.6)	52 (60.5)		
≥10 mm	40 (26.7)	6 (9.4)	34 (39.5)		
Number of aneurysms				0.048	0.826
< 2	135 (90.0)	58 (90.6)	77 (89.5)		
≥2	15 (10.0)	6 (9.4)	9 (10.5)		
Location of aneurysm				0.517	0.472
Anterior circulation	56 (37.3)	26 (40.6)	30 (34.9)		
Posterior circulation	94 (62.7)	38 (59.4)	56 (65.1)		
Surgical Timing				0.634	0.728
Ultra-early	107 (71.3)	44 (68.8)	63 (73.3)		
Early	19 (12.7)	8 (12.5)	11 (12.8)		
Middle-advanced	24 (116.0)	12 (18.8)	10 (14.0)		
Physical impairment				2.834	0.092
+	130 (86.7)	52 (81.3)	78 (90.7)		
–	20 (13.3)	12 (18.8)	8 (9.3)		
Pupillary dilation				6.243	0.012
+	62 (41.3)	19 (29.7)	43 (50.0)		
–	88 (58.7)	45 (70.3)	43 (50.0)		
Intracranial hematoma				2.451	0.117
+	104 (69.3)	40 (62.5)	64 (74.4)		
–	46 (30.7)	24 (37.5)	63 (25.6)		
Encephalocele				11.192	0.001
+	106 (70.7)	36 (56.3)	70 (81.4)		
–	44 (29.3)	28 (43.8)	16 (18.6)		
Hydrocephalus				0.900	0.343
+	70 (46.7)	27 (42.2)	43 (50)		
–	80 (53.3)	37 (57.8)	43 (50.0)		
Cerebral infarction				7.181	0.007
+	89 (59.3)	30 (46.9)	59 (68.6)		
–	61 (40.7)	34 (53.1)	27 (31.4)		
CVS				0.091	0.763
+	37 (24.7)	15 (23.4)	22 (25.6)		
–	113 (75.3)	49 (76.6)	64 (74.4)		
Intracranial infection				1.080	0.299
+	77 (51.3)	36 (56.3)	41 (47.7)		
–	73 (48.7)	28 (43.8)	45 (52.3)		
Refractory fever				21.366	< 0.001
+	75 (50.0)	18 (28.1)	57 (66.3)		
–	75 (50.0)	46 (71.9)	29 (33.7)		
Epilepsy				0.462	0.497
+	29 (19.3)	14 (21.9)	15 (17.4)		
–	121 (80.7)	50 (78.1)	71 (82.6)		

GCS, Glasgow coma scale; CVS, cerebral vascular spasm.

### 3.2. Construction of the nomogram risk prediction model

**Figure 1:**
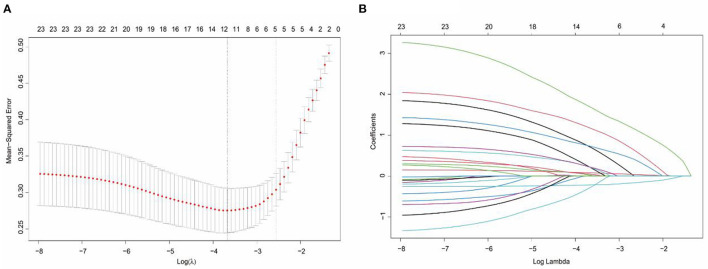
Selection of potential predictors using a LASSO regression model. **(A)** The optimal parameter (lambda) in the LASSO model was confirmed in the model by tenfold cross-validation of the minimum criterion. A dashed vertical line is drawn at the optimal value by using the smallest criterion (left dashed line) and one standard error of the smallest criterion (lambda.1SE) (right dashed line). **(B)** The model is optimal when λ increases to a standard error (lambda.1SE), and variables with nonzero coefficients were screened out as potential predictors. It effectively decreased the 21 influencing factors to 5 as potential predictors.

In the LASSO regression analysis, the optimal penalty coefficient (λ) was confirmed in the model by a tenfold cross-validation of the minimum criterion. The model is optimal when λ increases to a standard error (lambda.1SE), and variables with non-zero coefficients were screened out as potential predictors. This effectively decreased the 21 influencing factors to five potential predictors (Figures 1A, B). The five potential predictors were age, Hunt-Hess grade, GCS score, aneurysm size, and refractory fever.

**Figure 2:**
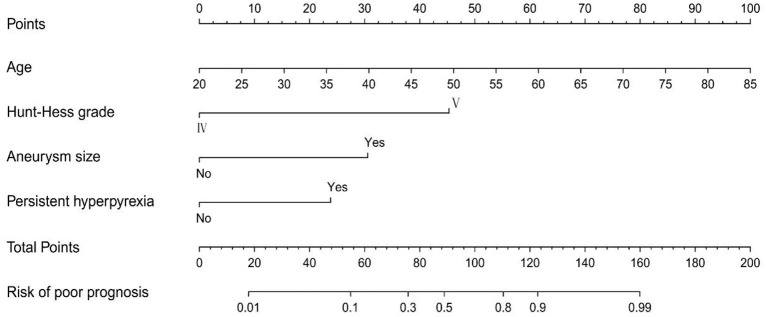
The nomogram model for predicting the risk of poor prognosis in patients with poor-grade aneurysmal subarachnoid hemorrhage following microsurgical clipping.

Based on the LASSO regression analysis, the five potential predictors were subordinated to univariate and multivariate logistic regression analyses to provide four independent predictors. As shown in Table 2, the four independent predictors were age (*OR*: 1.107, 95% *CI*: 1.056–1.116, *P* < 0.001), Hunt-Hess grade (*OR*: 8.832, 95% *CI*: 2.312–33.736, *P* = 0.001), aneurysm size (*OR*: 6.871, 95% *CI*: 1.907–24.754, *P* = 0.003) and refractory fever (*OR*: 3.610, 95% *CI*: 1.301–10.018, *P* < 0.001). Based on the results of logistic regression analysis, a risk nomogram determined by the four independent predictors was developed for predicting poor outcomes in patients with poor-grade aSAH undergoing microsurgical clipping (Figure 2).

**Table 2:** Univariate and multivariable logistic analysis.

**Variables**	**Univariate analysis**				**Multivariable analysis**			
	**B**	**Wald**	**OR (95%CI)**	***P*** **-value**	**B**	**Wald**	**OR (95%CI)**	***P*** **-value**
Age	0.059	12.609	1.061 (1.027–1.096)	< 0.001	0.102	17.502	1.107 (1.056–1.116)	< 0.001
Hunt-Hess grade	2.375	36.170	10.746 (4.956–23.298)	< 0.001	2.178	10.150	8.832 (2.312–33.736)	0.001
GCS	−0.622	29.581	0.538 (0.429–0.672)	< 0.001	−0.307	2.865	0.736 (0.516–1.505)	0.091
Aneurysm size	1.844	14.619	6.321 (2.456–16.264)	< 0.001	1.927	8.688	6.871 (1.907–24.754)	0.003
Refractory fever	1.614	20.144	5.023 (2.482–10.164)	< 0.001	1.284	6.075	3.610 (1.301–10.018)	0.014

GCS, Glasgow coma scale.

### 3.3. Validation of the nomogram risk prediction model

**Figure 3:**
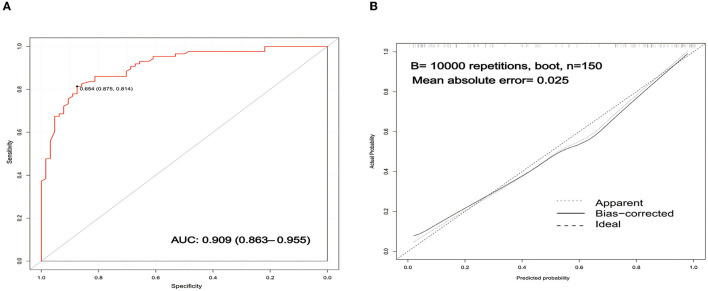
Validation of the nomogram. **(A)** ROC curves of the nomogram for predicting the risk of poor prognosis. The AUC of the nomogram was 0.909 (95% CI: 0.863–0.955). **(B)** Calibration curves of the nomogram for predicting the risk of poor prognosis. ROC, receiver operating characteristic; AUC, area under curve.

Receiver operating characteristic (ROC) curve analysis was conducted to assess the discrimination of the prediction model based on a risk nomogram. As shown in Figure 3, the area under the ROC curve (AUC) was 0.909 (95% CI: 0.863–0.955), indicating that the model had good predictive power. The results of the Hosmer–Lemeshow test showed that the deviation between the predicted risk values and the actual observed values of the columnar graph model was not statistically significant (χ^2^ = 8.931, *P* = 0.348), which implies that the columnar graph model has a good fit.

An internally validated bootstrap sampling method (1,000 times) was used to verify the nomogram model. The C-index value for the nomogram model was 0.9069 (95% *CI:* 0.9057–0.9086), implying that the model had good discriminatory and predictive power. The calibration curve is shown in Figure 3. The mean absolute error of the calibration curve was 0.025, and the slope of this curve was approximately one, indicating good consistency in the model for predicting poor prognosis.

**Figure 4:**
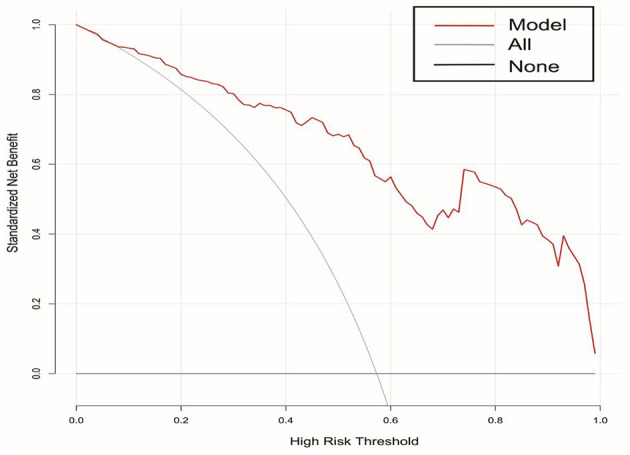
DCA curves of the nomogram for predicting the risk of poor prognosis in patients with poor-grade aneurysmal subarachnoid hemorrhage following microsurgical clipping. DCA, decision curve analysis.

A DCA curve was used to evaluate the clinical utility of the model. The results are shown in Figure 4. The abscissa and ordinate represent threshold probability and net benefit, respectively. The lines marked “None” and “All” represent the two extreme cases. The further away the model curve is from these two lines, the better is the clinical benefit of the nomogram. When the risk threshold was >0.02, the risk nomogram model predicting a poor prognosis exhibited better clinical utilization in the DCA curve.

## 4. Discussion

Poor-grade aSAH is a common neurosurgical cerebrovascular disease that threatens human health. Most patients with poor-grade aSAH experience direct damage to the brain tissue, secondary damage such as cerebral vessel spasm, secondary brain swelling, bleeding from reruptured aneurysms, and refractory fever (7, 12). Patient mortality has declined in recent decades with improvements in neurocritical care management, such as multimodal monitoring, the use of CTA, controlled decompression, and hypothermia, patient mortality has declined. Nevertheless, SAH remains a devastating disease with a high disability rate (13–16). Multiple risk factors affect the prognosis of patients with poor-grade aSAH. However, reliable and straightforward predictive models for prognosis prediction in patients with poor-grade aSAH who undergo surgical clipping are lacking. Therefore, it is important to focus on the prognostic outcomes of patients with poor-grade aSAH to improve their quality of life and survival. This retrospective observational study aimed to investigate the relationship between potential clinical risk factors and outcomes of poor-grade aSAH and to establish a nomogram to predict the risk of poor prognosis in patients.

In this study, 150 patients with poor-grade aSAH who underwent aneurysm clipping were enrolled to analyze the risk of poor prognosis. The results showed that age, Hunt-Hess grade, aneurysm size, and refractory fever were risk factors for predicting poor patient outcomes. Based on these four risk factors, a novel nomogram, visualization of the model, was established to help in the decision-making process to perform perioperative management and treatment. Through internal validation, we found that the nomogram had good calibration, predictive ability, and clinical applicability. Therefore, this predictive model could help predict poor outcomes and develop optimal strategies for patients with poor-grade aSAH undergoing aneurysm clipping.

Age plays an essential role in the clinical outcomes of patients with poor-grade aSAH. Recently, the number of patients with aSAH has been increasing as the population ages (17, 18). The treatment of elderly patients remains a clinical challenge. Over the last few decades, early surgical treatment has gradually replaced conservative treatment in the therapy of elderly patients with poor-grade aSAH, which provides a partial benefit in terms of prognosis (19, 20). Nevertheless, the death and disability rates remain high in patients with poor-grade aSAH. The present research indicates that age is a risk factor for poor outcomes in patients with poor-grade aSAH who are receiving microsurgical clipping and that the prognosis of patients tends to worse with increasing age. Goldberg et al. (21) reported that the risk of mortality and disability increased significantly with age in elderly patients with poor-grade aSAH. Elderly patients who commonly have underlying diseases, poor physical fitness, and varying degrees of brain atrophy, which make aging brain tissue less tolerant, are more susceptible to early brain injury, delayed cerebral ischemia and systemic complications (22). In addition, most aneurysms are prone to rupture because of their diminished vascular tolerance (23). Therefore, it is essential to prevent the occurrence of poor-grade aSAH in the elderly and to implement awareness of early prevention and detection.

The Hunt-Hess grading system, a simple and effective clinical grading scale for SAH, was designed to standardize the clinical assessment of SAH and facilitate patient management (24). Multiple studies have shown that the prognosis of patients with aSAH was significantly correlated with the Hunt-Hess grade (25, 26). In addition, our results showed that the Hunt-Hess grade was a solid predictor of poor outcomes in patients with poor-grade aSAH who underwent microsurgical clipping. Moreover, the risk of poor prognosis in patients with grade V disease was 8.832 folds that of patients with grade IV disease. Preoperative Hunt-Hess grading has the advantage of being more reflective of the patient's consciousness level than other grading systems. The Hunt-Hess score is closely monitored to reflect the severity of the patient's condition, and appropriate treatment measures have been developed to improve the prognosis.

Some investigations have identified a strong relationship between aneurysm size and the poor prognosis of aSAH (27, 28). Similarly, our study found that aneurysm size predicted a poor prognosis in patients with poor-grade aSAH who underwent clipping. Ruptured giant aneurysms frequently result in poor clinical condition up admission due to heavy bleeding, which increases the risk of surgery (29). In addition, these patients are more likely to experience severe brain damage and other complications. Previous studies have reported that giant aneurysms are a risk factor for rebleeding and poor prognosis in patients with aSAH (30, 31). However, some studies concluded that aneurysm size was not related to patient follow-up outcomes, including patients who received endovascular treatment and microsurgical clipping (32). Recent studies have investigated whether aneurysm size is a risk factor for poor prognosis, which may be a reason for the inconsistent results due to the choice of treatment modality. Currently, single surgical clamping is less effective for treating giant aneurysms. Hence, the development of an individualized, multidisciplinary, and thorough surgical strategy is critical to improve the prognosis of patients with poor-grade aSAH undergoing microsurgical clipping.

Fever is a relatively common complication in patients with poor-grade aSAH (33), which may be related to the influence of central, absorption, and infectious fever (34). Studies have reported that refractory fever can exacerbate neurological damage and is associated with increased survivor mortality, functional impairment and cognitive dysfunction (34, 35). Refractory fever was defined when the patient's temperature exceeded 38.3°C, simple physical and pharmacological cooling failed, and the fever was of unknown etiology. In the current study, we demonstrated that refractory fever was an independent risk factor for poor prognosis in patients with poor-grade aSAH who underwent surgical clamping. Brain and body temperatures are closely linked, and studies have noted that patients with a *T*_delta_ (brain temperature subtract body temperature) of < 0 have a poor prognosis (36). Furthermore, excessive body temperature, leading to altered temperature rhythms in patients with poor-grade aSAH, may be a contributing factor. Recently, targeted temperature management (TTM), a treatment strategy for critically ill patients, has been proposed to reduce the secondary neurological damage of poor-grade aSAH (37, 38). Temperature changes contribute to prediction of patient prognosis, particularly in patients with refractory fever. Therefore, a reliable model based on refractory fever for predicting patient prognosis and providing strategies for perioperative treatment would benefit patients and minimize their risks.

Nomograms have been studied in the field of aSAH; however, there are few studies on poor-grade aSAH (39–41). To the best of our knowledge, no nomogram studies have been performed on patients with poor-grade aneurysms who underwent surgical clipping. To our knowledge, this is the first accurate prediction model based on age, Hunt-Hess grade, aneurysm size and refractory fever. Mortality and disability remain important issues in patients with poor-grade aSAH. The development of predictive models can serve as guides for patient management. In the current study, we identified four essential clinical predictive features: age, Hunt-Hess grade, aneurysm size, and refractory fever. The nomogram is a more practical visualization tool than other traditional tools. Through internal validation, we found that this nomogram was highly distinguishable, consistent, and clinically useful.

This study has some limitations that require attention. First, although the risk model showed excellent accuracy after internal bootstrap validation, it still lacks external validation. Second, we did not record basic vital information using devices such as cardiac monitors, ventilators, or pressure sensors in the ICU, which may have provided a deeper understanding of the patient's condition. Lastly, this was a single-center retrospective investigation with a small sample size, and the results may have been biased.

## 5. Conclusion

Several risk factors affect the prognosis of patients with poor-grade aSAH, and there is an interplay between these factors. In this study, we identified four essential clinical predictive features: age, Hunt-Hess grade, aneurysm size, and refractory fever by LASSO, univariate, and multifactorial logistic regression analyses. In addition, we developed a nomogram using these four predictors. Based on internal validation, the model has good accuracy and clinical utility in helping clinicians assess the prognosis of patients with poor-grade aSAH who underwent surgical clamping.

## Data availability statement

The original contributions presented in the study are included in the article/supplementary material, further inquiries can be directed to the corresponding authors.

## Ethics statement

The studies involving human participants were reviewed and approved by Ethics Committee of Wuxi Clinical College of Anhui Medical University. Written informed consent to participate in this study was provided by the participants' legal guardian/next of kin.

## Author contributions

Conceptualization: ZZ, JC, and YW. Methodology and software: ZZ. Validation: ZL, HY, and ChuZ. Formal analysis and visualization: ZL. Investigation and supervision: HY. Resources: ChuZ. Data curation: CheZ. Writing—original draft preparation: ZZ and JC. Writing—review and editing: JC and YW. Project administration: JC. Funding acquisition: YW. All authors have read and agreed to the published version of the manuscript.
